# Dr. Barker’s Case of Double Brain

**Published:** 1844-01-27

**Authors:** 


### DR. BARKER'S CASE OF DOUBLE BRAIN.

On October 17th my attendance was requested on Mary Nelson, ætat. 32, who on that day was seized with labour-pains of her seventh child. On arriving at the house, and making an examination, I found the os uteri fully dilated, and the pains strong and constant. The vertex presented, with the face towards the left ilium. Upon the head descending into the cavity of the pelvis I became aware of the existence of a tumour, the nature of which I could not at first distinguish. The pains, however, soon expelled the child, when I found the tumour to be attached to the occipital bone, and of the size of a large orange, of a soft nature, and totally devoid of hair, although hair was plentiful upon the head. In connection with this the child had spina bifida of the third, fourth, and fifth dorsal vertebræ, and the right foot was clubbed. The other organs were normal. The child was seized with convulsions immediately after its birth. These continued, at intervals, until its death, which took place on the following evening. Permission being given, I opened the tumour on the morning afterwards, and found it to contain a perfectly developed brain, surrounded by its ordinary membranes, and connected with the posterior part of the cerebrum within the skull, through a circular opening of about an inch in diameter, just above the occipital protuberance, and having, also, a distinct connection with the spinal cord, the nerves being given off in the usual manner.—*London Lancet*, Nov. 11th, 1843.

